# PAtCH: Proactive Approach to Circumvent Holes in Wireless Sensor Networks

**DOI:** 10.3390/s23187862

**Published:** 2023-09-13

**Authors:** Moyses M. Lima, Eduardo D. Sardinha, Leandro N. Balico, Horacio A. B. F. Oliveira

**Affiliations:** 1 Retail Management System Technical Group, SIDIA R&D Institute, Manaus 69055-035, Brazil; 2 Application Technical Group, SIDIA R&D Institute, Manaus 69055-035, Brazil; eduardo.sardinha@sidia.com; 3 Department of Computer Science, Federal University of Roraima, Boa Vista 69310-000, Brazil; leandro.balico@ufrr.br; 4 Institute of Computing, Federal University of Amazonas, Manaus 69080-900, Brazil; horacio@icomp.ufam.edu.br

**Keywords:** routing holes, local minimum, geographic routing

## Abstract

The occurrence of hole regions in Wireless Sensor Networks is a significant challenge when applying a greedy technique in a geographic routing approach. The local minimum phenomenon is commonly attributed to physical obstacles, energy depletion of the nodes, failures in communication between neighbors, or even the incorrect deployment of the nodes in the sensing field. To address the problem of hole regions, most approaches choose to abandon the traditional greedy forwarding mechanism to temporarily adopt the well-known perimeter routing scheme applied to nearby nodes or along the edge of a region of a hole. However, this mechanism does not satisfy the network load balance requirement, because it imposes too much traffic to the nodes in the hole’s edge, making them overloaded when compared to other network nodes more distant from holes. In this work, we propose a novel location-free geographic routing technique called PAtCH (Proactive Approach to Circumvent Holes in Wireless Sensor Network) to avoid routing holes in WSNs. Our solution can circumvent hole regions and create routing paths toward the destination. We consider that our sink has a higher communication range, and the Received Signal Strength Indicator (RSSI) is used to assist the construction of the routing paths. Our results show the efficiency achieved by our proposed solution in scenarios with hole regions, also maintaining all the benefits of a classic greedy forwarding technique.

## 1. Introduction

Wireless Sensor Networks typically consist of a collection of sensor devices that are tactically placed in a designated area of concern. Their purpose is to supervise the physical and chemical environmental circumstances in a variety of locations [1]. Due to the singular nature of projects involving WSNs, most of the implementations have limiting characteristics, such as the residual energy of the nodes and bandwidth. Thus, mechanisms for optimization of such resources, aiming to enable the network to operate efficiently and correctly, are entirely necessary and preferable. In addition, WSNs can be used to monitor different types of information, such as relative air humidity, movement of targets (animals, people, objects), variations in lighting, atmospheric pressure, and soil composition, as well as characteristics, such as speed; even the size of objects are the subject of applicability for such networks [2].

During a WSNs’ operation, one of its most important steps is the transport of collected data over the network. In this way, sensor node-gathered information must be correctly routed to a treatment plant, usually called a sink. Generally, the data dissemination process is carried out using a multi-hop message forwarding scheme towards the sink, already equipped with mechanisms for processing and handling the information [3]. According to Akyildiz et al. [1], it is possible to classify WSN routing approaches into two categories: (*i*) single-hop approaches and (*ii*) multi-hop approaches. In the first category, regular nodes send their information directly to the destination, simplifying the implementation of packet delivery solutions. However, the incorporation of a wireless communication device with long-range capability, which is typically associated with high power consumption and hardware expenses, is deemed imperative for the implementation of this methodology. For the multi-hop approach, regular nodes send their information to the intended destination using short-range communication established with one or more neighbors in the intermediate location.

Routing is the process of choosing a valid path between source and destination, according to the received transmission requested. In the domain of wireless sensor networks, there is no pre-defined infrastructure since the nodes that are placed in the surroundings to be supervised are susceptible to experiencing defects and limitations in their energy resources. For this reason, routing protocols developed for WSNs need to meet a balanced set of requirements, such as autonomy, energy efficiency, scalability, resilience, heterogeneity (in relation to sensors), and mobility [4]. Thus, providing the ideal conditions for efficient traffic of collected information should be part of the designers and developers efforts for improving such routing protocols. According to Wang et al. [5], improving the process of data transmission between the nodes can significantly reduce the total energy utilized by the network, thereby improving the network’s lifetime and ensuring sustained connectivity in the network.

**Figure 1:**
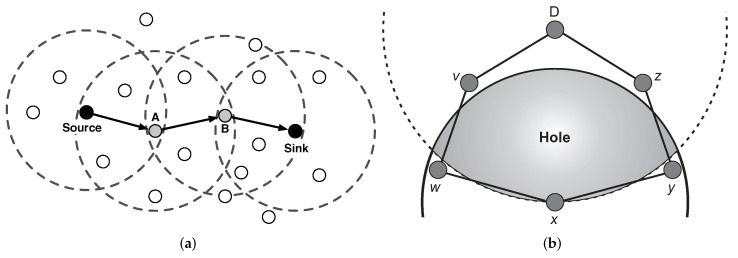
(**a**) Greedy path computing using geographical approaches [9]. (**b**) Local minimum phenomenon: a routing hole between ***x*** and **D** [10].

Several general purpose routing algorithms have been proposed for WSNs. Among these solutions, we can highlight the ones that exploit geographic information, called Geographic Routing Algorithms, which are an alternative to implement data transport in such networks easily [6,7,8]. These approaches use the position of the network nodes to create paths towards the final destination of the messages. Each node only needs to know the location of its neighbors one hop away to compute route paths in a distributed fashion. In this case, the greedy forwarding mechanism has been widely adopted by geographic routing solutions. Such approaches perform packet routing by forwarding the messages to the neighbors geographically closest to the destination [9]. Figure 1a shows the operation of the greedy forwarding mechanism. In this scenario, the Source node selects node **A**  because it is geographically closest to the destination **Sink**. The same happens with node **A**, which chooses node **B**, geographically closest to the **Sink**. Finally, node **B** forwards the received packet to the final destination, in this case, the **Sink** node.

The literature highlights the adoption of geographic routing techniques in WSNs, as such techniques offer benefits such as improved scalability, greater energy efficiency, and an economical approach to building data forwarding routes. However, the correct operation of such algorithms requires the prior execution of a localization system to obtain nodes’ location information, which in these cases are not always available. In addition, the occurrence of routing region holes during the forwarding process of messages to their final destinations is still considered one of the significant challenges to be solved for the efficient operation of such approaches. In this work, we consider hole regions as a specific category of the local minimum phenomenon, widely discussed in [11,12] and also depicted in Figure 1b. In the figure, it is possible to observe that the greedy forwarding mechanism will fail since **x** has no eligible neighbors at the next hop, i.e., there is no hop geographically closer to  **D**, in this case, the final destination of the flow. This phenomenon, in practical terms, can occur due to physical obstacles, energy depletion, communication failures among elected neighbors, and deployment in the sensing field.

Hole regions emerge as a distinct category of problems associated with both the local minimum phenomenon, as extensively discussed in [13], and physical obstacles, exhaustion energy, failures in communication between elected neighbors, and incorrect implementation in the field or area of interest to be sensed. One of the most widely used alternatives to deal with hole regions problem is to avoid the use of traditional greedy routing mechanisms and adopt the well-known perimeter routing scheme. This scheme is applied to the nodes near or along a hole’s region, and it also involves knowledge about the hole boundaries, as well as requiring the maintenance of the paths on the borders of such holes. However, the perimeter routing strategy imposes too much traffic on nodes located at these hole edges, leading to a fast energy depletion of such nodes and increasing the size of the holes as a consequence [14].

Hole regions can affect the performance of WSNs since they reduce the reliability of routing solutions, introduce deformations on network topologies, interrupt communication between nodes or even parts, and increase the severe workload on nodes located in the neighborhood of a hole. Such characteristics should be considered when adopting geographical approaches since it requires the use of more efficient techniques for detecting and avoiding hole regions. Another essential aspect to recognize is that, in most geographic solutions, sensor nodes are equipped with a position tracker or can use localization algorithms. However, providing nodes with positioning hardware, such as a GPS, can cause significant additional costs. Furthermore, localization algorithms tend to deplete the power of the sensor node batteries, causing failures on the network. Thus, taking all these factors into account, our proposed PAtCH algorithm considers that the sink node has greater communication capability, allowing it to, in a single hop, reach every network node in the initial query [15]. Reply packets from regular nodes are forwarded to the sink using multi-hop paths computed by our novel location-free geographic routing technique, which uses the RSSI (Received Signal Strength Indicator) values to choose the hop geographically closest to the sink and to avoid hole regions during the routing process.

The motivation for the PAtCH algorithm came from the ATTO (Amazonian Tall Tower Observatory) project [16], implemented in the Amazon region of Brazil, which consists of a 320-m-high tower embedded in the middle of the forest. Its privileged physical structure and large capacity communication devices, already used by the project, perform different types of measurements related to the Amazon, such as micro-meteorological and atmospheric chemical variable, precipitation, temperature and wind profiles, water and energy fluxes, radiation fluxes, soil temperature profiles, and soil heat fluxes and turbulence components.

The remainder of this paper is organized as follows. In the next Section, we describe the related work. In Section 3 we describe the PAtCH algorithm, which is evaluated in Section 4. In Section 5, we briefly discuss the applicability, advantages, and limitations of the proposed solution. Finally, in Section 6 we present our conclusions and future directions.

## 2. Related Work

Geographic routing poses a persistent challenge in Wireless Sensor Networks (WSNs) owing to its consequential benefits in the realm of energy savings. Additionally, it can provide high delivery rates in a dense network. The central strategy of these algorithms is based on reliance on geographic location information (i.e., latitude, longitude) of the sensor nodes to create packet flow paths to the desired destination [17,18,19]. In traditional geographic routing, the greedy forwarding mechanism is responsible for packet forwarding and is performed as follows: each node needs to know the position of its one-hop distance beyond the location of the final destination of the packets. With this information, the source node will choose a neighbor that is geographically closer to the destination, usually a sink [20]. This position information regarding the destination of the packet is usually appended to the packet header so that the intermediate neighbors can understand and learn about the final packet destination.

Although the greedy forwarding mechanism is useful and provides scalability with high delivery rates, the occurrence of holes or regions where multi-hop communication fails is still a challenging limitation faced by most traditional routing protocols [21]. Such a phenomenon, also known as the local minimum phenomenon, is a classic problem for geographic routing approaches and occurs when no eligible neighbor is geographically closer to the final destination  [22,23]. Among the most known strategies used in the design of solutions to the local minimum problem, we can highlight the use of solutions to detect and recognize network border nodes and hole regions.

One of the first attempts to extend geographical routing functionalities through the improvement of the packet-forwarding process in scenarios with the presence of holes was GPSR (Greedy Perimeter Stateless Routing), proposed by Karp et al. (2000) in [10]. This routing scheme uses the positions of the regular nodes and the final destination of the packets to make greedy routing decisions, based on the neighbors’ information to a single hop away. The operation of the algorithm takes place in two distinct stages: (*i*) the use of a greedy routing mechanism and (*ii*) perimeter routing, used in regions where the first stage fails. When the algorithm is executed, when a packet reaches an area where greedy routing is not possible, the algorithm starts to operate in perimeter routing mode. In this way, every time the packet encounters an edge or an optimal path, the transfer of packets follows the surface of the perimeter, alternating between the edge nodes in a strategy known as the right-hand rule.

The occurrence of hole regions in WSNs still emerges as a challenge lacking an effective solution since most of the solutions implemented follow the classic combination of hole detection and hole bypassing. Such a combination can potentially contribute to factors that cause an increase in the size of the network holes and also its impacts [23]. Kanno et al. (2009) [24] investigated the occurrence of hole regions in WSNs as a coverage problem. They considered the availability of a network communication graph and proposed a technique for detecting coverage holes. In this technique, sensor nodes that connect the coverage holes and process information incorporated in the graph are identified and categorized to obtain a second planar graph that is equivalent to a hole and to maintain the quantity and position of the holes. Finally, a planar simplicial complex called maximum simplicial complex, which contains the information about the hole regions, is implemented.

An algorithm that detects routing holes and calculates the size of the area occupied by this void area has been proposed in [25]. In this approach, called the Wireless sensor Hole Detection algorithm (WHD), the sensors are categorized into cells or Regions Of Interest (ROI) that contribute to increased network lifetime and discipline the communication of the regular nodes with the sink. In [26], a routing solution for Wireless Sensor Networks, composed of two algorithms, was proposed and discussed. The Hole Alleviation-Energy Conditioned Mean Absolute Error (HA-ECMAE) and Hole Alleviation-Energy Conditioned Mean Absolute Error Two Hop (HA-ECMAE2H) can handle the energy imbalance caused by the occurrence of routing holes during the packet-forwarding process in multi-route and multi-hop scenarios.

A congestion control approach for geographic routing algorithms was proposed in [27]. In this particular scheme, every node carries out regular monitoring of its own receiving queue length and remaining energy level, and during each step the node checks for the proximity of a failed node, also known as a routing hole, and increases its distance from the sink to circumvent the hole and keep forwarding packets to the sink. In [28], the modified Greedy Perimeter Stateless Routing (GPSR) algorithm was proposed to determine the optimal path based on energy usage, and it avoids malicious activity. During the process of forwarding packets to the sink, considering the presence of routing holes, the algorithm can forward packets reliably by directing data through incident surveillance nodes to event-detecting networks, thereby producing an energy-efficient routing mechanism.

In [29], the HPS (Hole Plastic Scheme) is proposed. Its key idea is to adaptively fill the concave area of the hole region with potential stuck nodes, based on its one-hop information from neighbors. Nodes located in the concave area of the hole are marked and do not participate in the data routing process unless a source or a destination is located in the hole region. At the end of the filling process, the data that potentially flow into the hole region will automatically bypass the hole without the need for packet routing additional tasks. According to [30,31], hole regions may also be associated with unbalanced deployment. The RNGHAR (Hole Avoiding Routing protocol) algorithm [22] uses an RNG (Relative Neighborhood Graph) hole modeling scheme to collect hole region information to detect the position of the hole region in advance and construct an avoiding path around the hole region. Nodes located at the edge will use the hole information to prevent packets from following the natural flow, i.e., entering the hole, and to choose a shorter route to the final destination.

A geographic routing protocol for WSNs that implements a geolocation system that is aware of geographic coordinates, called SGFTEM, is presented in [32]. In this approach, each node is aware of its respective neighbors, and such information is updated at each hop, while forwarding packets to the sink. Furthermore, each node can decide how to forward packets to a neighbor, considering the node’s throughput and the distance to the destination. Another geographic routing protocol based on Proximal Policy Optimization was presented and discussed in [33]. The main goal was to give nodes the ability to choose routes that maintain balance while forwarding packets to the sink.

To fill communication voids, which can be formed by the random distribution of sensor nodes in the grid, the BVR-VCM (Bypassing Void Routing based on Virtual Coordinate Mapping) [34] algorithm is able to form a virtual circle from the coordinates of the edge nodes. The goal is to convert a random structure of nodes that form a possible hole into a regular structure by mapping the coordinates of the edge nodes to a virtual circle. This process allows the data packets to be routed in paths around the hole according to the virtual coordinates of the edge nodes. Recently, a routing scheme that considers the hole-covering parallelogram and the hole view angle of a specific node was proposed in [35]. In this approach, data packets can be routed along a new escape route that circumvents the hole and flows to the destination. Finally, the LRS (Novel RSSI-based Algorithm), which uses RSSI values as a metric to generate paths around holes, even when the physical location of the nodes are unknown, was proposed in [36]. In this algorithm, the stuck node selects an alternative neighbor towards the sink based on the extra information stored during the distance estimation. Like our PAtCH algorithm, LRS also considers the existence of a long-range sink so that, in a single hop, it is possible to reach the whole network.

In Table 1, we classify and compare the aspects related to the dependence on location systems, packet flow during forwarding to the sink, and, finally, whether the approach implements mechanisms to avoid the region of holes before forwarding the packets. The first group includes methods that do not rely on location systems, such as GPS or virtual coordinates, to determine the next packet-forwarding hop. In the second category, the algorithms are capable of generating alternate paths for forwarding packets, resulting in balanced flows while optimizing energy resources. Finally, in the third category are techniques that avoid the hole region, preventing node overload in this location. Our PAtCH algorithm supports blind packet transmission and can deal with routing holes while offering multipath data aggregation without requiring sensor node position information to pick the next hop.

With the exception of LRS, PAtCH differs from all of the aforementioned solutions due to its hole discovery mechanism, which does not rely on positioning systems, whether in virtual or real coordinates (such as GPS). Furthermore, the choice of the next-hop eligible neighbor uses the RSSI values reported from the sink node and regular nodes. Thus, LRS is similar to our approach since it also uses RSSI values as the basis for forwarding packets to the sink and chooses the greedy neighbor to the forward packet process. Our proposal is also similar to the methodology used in the GPSR protocol since we are proposing the creation of greedy routing flows around the hole region. However, it is important to notice that our current proposal differs from previous attempts because it does not depend on any sensor nodes’ computed localization information or even equipped GPS on any nodes. Furthermore, we find evidence that, in all the previous approaches before this work, the size of the border of the hole region can increase and compromise the data forwarding process to the final destination because the stuck node must be known in advance. Finally, our PAtCH algorithm is able to make better-quality geographic decisions since the packets forwarded to the sink can avoid hole regions.

In this work, we propose a novel location-free geographic routing approach that avoids routing holes through a technique that uses the RSSI values of both the sink node and the regular nodes to create alternative paths for the flow of data packets, called PAtCH (Proactive Approach to Circumvent Holes in Wireless Sensor Network). PAtCH can create routes around a region of holes without the need for expensive computations in the stuck node. In this way, dead-end nodes (nodes in a hole region) are not activated by our algorithm since the RSSI values, propagated in the initial stage of the algorithm’s operation, can be used as the knowledge base for the creation of the flow paths in the neighborhood of a hole region. The PAtCH algorithm is designed to be as reliable as a classical geographic algorithm, including high delivery rates and energy savings during the packet-forwarding process, without requiring any virtual coordination assignment or any other costly localization system.

**Figure 2:**
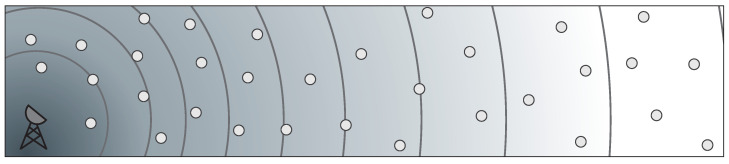
RSSI values decreasing according to the message propagation on the network. Based on [43,44].

PAtCH considers that the sink node is equipped with a communication device powerful enough to reach all nodes deployed in the grid (sensing environment) in a single communication hop. The sensor nodes will receive the sink messages with different RSSI levels in such a way that most distant nodes receive the message with lower signal strength due to propagation issues (i.e., signal attenuation), as depicted in Figure 2. The efficient use of RSSI as a metric for localization systems has already been shown and discussed in [37,38,39,40]. The received signal strengths can be converted to estimated real distances, as already observed in  [41,42]. This is a reasonable assumption, as discussed in Section 5. In addition, to simplify the understanding, the term distance estimations will be used instead of received signal strength.

## 3. PAtCH Algorithm

**Figure 3:**
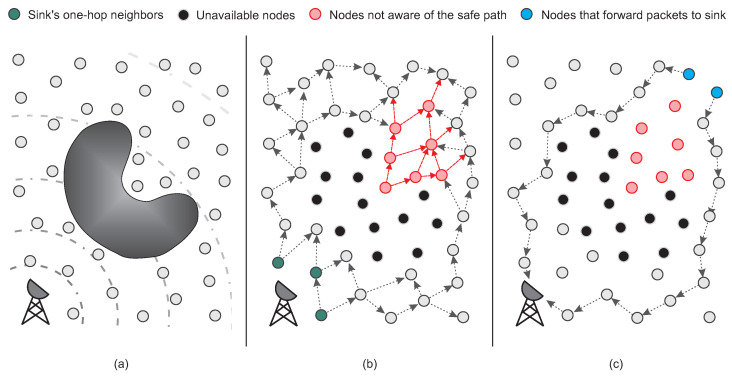
(**a**) Sink node sends a query to all nodes in a one-hop communication. (**b**) A node that received the sink query and is a one-hop sink neighbor will then propagate such information during the distance estimation step. (**c**) Chosen nodes will start the forwarding step. Neighbor nodes will use the secure path to avoid the region of holes during the packet-forwarding process.

In the PAtCH algorithm, the RSSI values, disseminated by the sink node, are used both to generate flows to return the data packets back to the sink and to estimate the distance between a transmitter and a receiver, as depicted in Figure 3a,b. Our technique allows each node to choose a neighbor outside the hole region and, at the same time, be geographically closest to the sink node, as depicted in Figure 3c. This is possible because, after the propagation of the first query from the sink, regular nodes begin an announcement process of their estimated distance to the sink node to one-hop neighbors. Thus, the choice of which neighbor can forward the packet is computed based on the distance information obtained during the dissemination stage. An additional field is added to the packets sent during the dissemination stage of estimated distances. Such a field is used to allow the sink neighbors, i.e., nodes one hop away from the sink node, to be used as a reference to create secure paths for the flow of the packets. This extra information is used by the regular nodes to find a route toward the sink.

**Figure 4:**
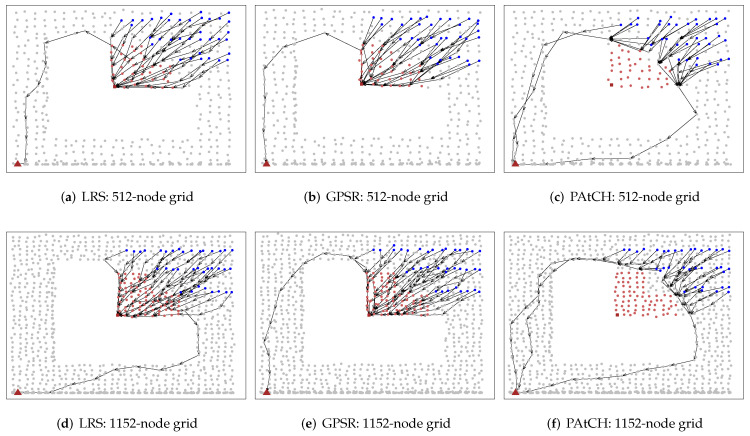
Packet flow trees generated by simulations.

To evaluate and compare the flow paths generated by both approaches, Figure 4b,c shows the route graphs generated during the simulations. In those figures, it is possible to note that the routing paths produced by PAtCH possess the ability to circumvent the hole while efficiently transmitting packets towards the sink.

The full implementation of the PAtCH algorithm is shown in Algorithm 1. It starts when the sink node propagates a query to the entire network in a single hop, as shown in Figure 3a. When receiving the query, each node estimates its distance from the sink node using the RSSI values and checks if it is a real neighbor of the sink node, i.e., if it is a hop away from the sink, as also depicted in Figure 3b. If so, such a node will add extra information in its advertisement packet that will be used in the process of forwarding data back to the sink node.

### PAtCH Operation

At this point, it is imperative to consider two crucial pieces of information: First, the signal power of the RSSI of the sink perceived by the regular nodes is used to calculate the distance from regular nodes to the sink and also the time that each node will wait before forwarding its packets. Secondly, the nodes that are one hop away from the sink, i.e., nodes that have the sink as a direct neighbor, will initiate a connectivity strike, thereby providing an alternative pathway to a node that may be potentially trapped in a routing hole.

The node then sends an advertisement packet to all of its neighbors containing its estimated distance, already computed in the previous stage, from the sink and the route information that will be used to create a connected path towards the sink. A startup timer is performed and started on the nodes that will participate in sending data. The nodes that will participate in packet forwarding are called safe hops (nodes in red in Figure 3a,b) and refer to nodes that receive data packets in multiple hops from the immediate neighbors of the sink node. Such packets contain their distances to the sink. This time unit is defined according to the received sink’s RSSI values by each node. When the timer expires, a reply packet is assembled containing both the safe hop information and the node’s distance to the sink.

**Algorithm 1** PAtCH

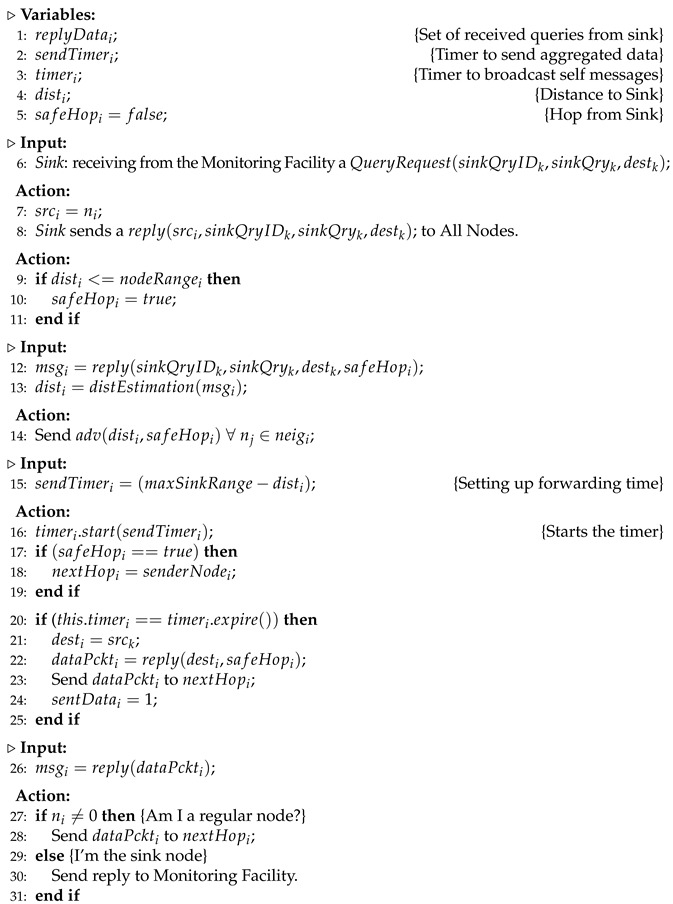



PAtCH considers the valid path tree constructed during the distance estimation process through the advertisement messages. To evaluate our PAtCH algorithm, we compare its operation with the classic greedy forwarding mechanism that focuses on sending packets to a node geographically closest to the sink. However, the sending packet process obeys the safe hop criteria (as already explained) and not the classical greedy forwarding approach, also shown in Figure 3c. This process repeats until the packet reaches the sink node, in which case the received data is sent to a central monitoring facility.

Differently from the current solutions, in our approach nodes in the hole region do not need to create routing tables or make any costly computation to detect or bypass the hole area since the generated flows from the sink node will naturally surround the hole. In the GPSR approach, the nodes at the edge of the hole are overused. This repetitive behavior can lead to the energy depletion of these nodes. In this way, our novel location-free geographic routing scheme is more effective in avoiding hole regions and provides geographic routing paths compatible with the classic greedy forwarding mechanism regarding efficiency.

## 4. Performance Evaluation

We evaluated the performance of the PAtCH algorithm by comparing it to the geographic approach called LRS (Long-Range SINK) [36] and the GPSR (Greedy Perimeter Stateless Routing) [10] algorithm, a classical approach for detecting and bypassing holes that uses perimeter routing to forward packets towards the sink. Both LRS and GPSR approaches are able to detect and bypass routing holes. We intend to validate our novel location-free geographic scheme, which requires neither location information nor virtual coordinates.

### 4.1. Methodology

The performance evaluation was performed through simulations using Sinalgo [45]. This simulation environment, built in Java, provides the necessary framework for simulating and testing distributed algorithms and is focused on the validation and analysis of network algorithms. In addition, it provides view-passing messages from the network without considering the underlying layers. Sinalgo can execute complex simulations in feasible execution time and is also a platform-independent tool. The visualization of hardware devices in Sinalgo is similar to that of real devices, such as TinyOS, where a node can forward messages to specific neighbors or all neighbors. It is also possible to implement reactions to incoming messages and to configure timers for scheduling future actions. Finally, by ignoring the physical layer this framework presents an important level of scalability and can simulate up to one hundred thousand nodes per project [46,47].

The simulations were performed on a 200 × 200 m${}^{2}$ sensing field, obeying a disturbed grid and keeping the node density in 0.03 nodes/m${}^{2}$. We also consider high densities where a large number of nodes have been deployed in a small area [48]. Regarding the results obtained, curves represent average values, while error bars represent confidence intervals for 95 % confidence from 33 different random seeds. The simulation parameters are based on the MicaZ sensor node, which operates in accordance with the 802.15.4 standard, and the default values are shown in Table 2.

We assume that the location of each node is disturbed by a random zero-mean Gaussian error and the nodes occupy the sensor field without forming a regular grid, while the sink node was positioned in the lower left corner. We also consider that the RF Received Signal Strength (RSSI) indication can be read directly from the nodes, as discussed in [49]. RSSI inaccuracy was simulated by considering that each range sample is disturbed by a Gaussian distribution with the actual distance as the mean and a percentage of this distance as the standard deviation [38,50].

During the PAtCH algorithm operation, each node assembles a single packet containing its estimated distance from the sink. This is done shortly after receiving the query with higher signal strength from the sink node. When the node timer expires, it sends its data packet only once. After that, packets generated and sent by neighbors follow the proposed location-free geographic scheme highlighted in this work. To evaluate the avoidance of the hole region, a random set of nodes located in the upper right corner of the grid was selected in each simulation cycle. The sink node was deployed on the opposite side, i.e., on the lower left corner of the grid. The key idea was to observe and analyze the flow of packets from the chosen regular nodes towards the sink. In this way, it was possible to evaluate the viability of our approach regarding the avoidance of holes.

A region of holes was designed and implemented for the execution of the experiments. The simulation grid was configured by inserting a concave irregular *L*-shaped polygon at the center, as shown in Figure 3a. The internal dimensions of the hole have been defined such that the nodes inside this area can not communicate with nodes outside. In addition, nodes on one side of the hole can not communicate with the nodes on the other side. This configuration can be considered as the worst scenario since a hole based on a circle does not always create a routing hole.

### 4.2. Network Density

**Figure 5:**
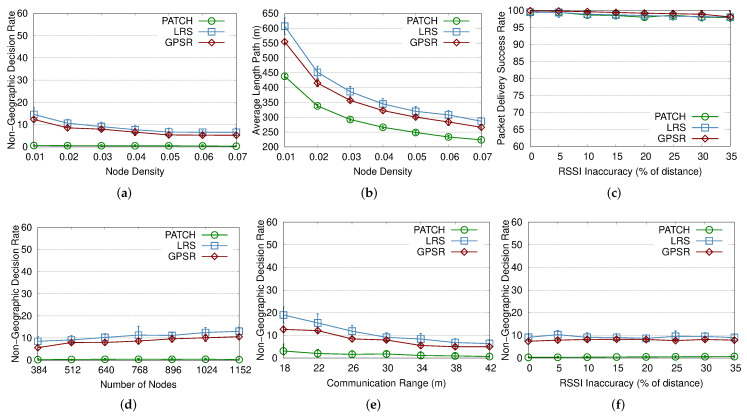
Performance evaluation of the PAtCH algorithm, with emphasis on non-geographical choices.

The impact of network density was evaluated, starting with a low density of 0.01 nodes/m${}^{2}$ and increasing this parameter to 0.07 nodes/m${}^{2}$. Increasing this value results in a larger number of neighbors, and consequently more reference points to be used in choosing the next forwarding hop. The choosing pattern of the next hop away observed in the PAtCH algorithm can make efficient geographic choices toward the sink while avoiding the hole region, as we can see from Figure 5a,b. Those parameters also show the percentage of wrong decisions when choosing the next hop node towards the sink (i.e., the selected node is not the closest to the sink node). In this sense, even at higher densities, our PAtCH algorithm was able to outperform the GPSR algorithm. At smaller densities and keeping the same communication range, all approaches face the problem of the low amount of neighbors. However, the PAtCH algorithm still works and can operate efficiently without the need to perform additional calculations or extra loops to exit the hole region, because the natural packet flow does not enter the hole region at all.

### 4.3. RSSI Inaccuracy

**Figure 6:**
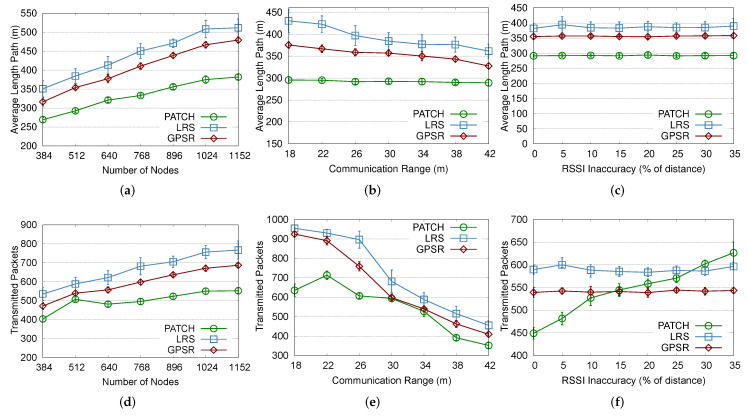
Performance evaluation results of the PAtCH algorithm, with emphasis on transmitted packets.

The RSSI inaccuracy was evaluated by increasing this imprecision from 0% to 35% of the real distances between nodes. As this value increases, there are more neighbors and, presumably, more reference points for sending and receiving packets. The LRS and GPSR approaches are not highly affected by the increased RSSI impression, as shown in Figure 5c,f and Figure 6c,f. However, as depicted in Figure 6f, we can see that the PAtCH algorithm is affected due the distance estimation when considering the number of packets transmitted towards the sink. It is important to remember that our novel location-free geographic routing mechanism depends on the values reported by both the sink and the regular nodes. Thus, the imprecision of the RSSI in the distance estimation process may affect the next hop decisions. Despite this result, our PAtCH algorithm can deliver more than 98% of the packets, is more effective in choosing the neighbor geographically closest to the sink, and, lastly, provides better paths toward the sink.

### 4.4. Network Scale

The scalability was evaluated by increasing the number of nodes in the network from 384 to 1152, with a constant density of 0.03 nodes/m${}^{2}$ and a communication range of 30 m, and the sensor field was resized according to the number of sensor nodes in each simulation. In all cases, the algorithm performance of the approaches are not highly affected by the number of nodes, keeping the rate close to 100% in all simulations. Regarding this result, in Figure 5d, we can see that our PAtCH algorithm can make better geographical decisions at each step since packets forwarded to the sink do not enter the hole region. It is important to highlight that our geographic approach was able to make 100% effective geographic choices, considering the proposed scenario already shown in Table 2. This feature results in smaller and more efficient routing paths to the sink, as shown in Figure 6a, since the packet does not enter the hole, as depicted in Figure 4. Differently from PAtCH, the LRS and GPSR algorithms have a lower amount of good geographical decisions of the next hop, resulting in larger flows towards the sink, as also shown in Figure 5d and Figure 6a.

### 4.5. Communication Range

We evaluated the impact of the communication range of the nodes by increasing this parameter from 18 m to 42 m. When the communication range is short, the number of neighbors reached by this node is smaller, which reduces the number of path possibilities, especially for the GPSR algorithm since it is affected by the number of neighbors in multiple hops. In this case, the next hop choosing can be less effective and may lead to larger paths toward the sink. As we can see in Figure 5e, PAtCH is still able to make better geographic decisions at each step than both the LRS and GPSR algorithms, even when we increase the communication up to 42 m. We can also see that the choice of the best routing paths to the sink also influences the average size of these paths, resulting in a smaller number of hops built around the hole. As we can see in Figure 6a, even considering a small number of neighbors, the PAtCH algorithm can generate better packet flow trees than the LRS and GPSR approaches. In addition, the PAtCH algorithm can avoid unnecessary packet exchanges in the hole region, resulting in a small number of transmitted packets in the area, thus not contributing to the increase of the void region problem, even when the number of neighbors is greater than 40.

### 4.6. The Edge of the Routing Hole

**Figure 7:**
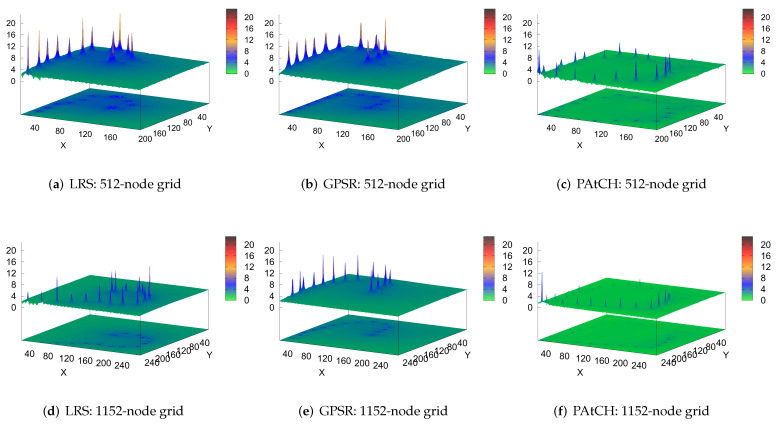
Performance evaluation results according to the transmitted packets when considering the position of the nodes in the grid.

We also evaluate the impact of the excessive utilization of nodes located on the edge of the hole. In both the LRS and GPSR algorithms, all messages are already entered inside the hole, leading to the more frequent usage of the node that is trapped within the hole. The key feature of geographic routing approaches is the choice of a neighbor geographically closer to the final destination of the packets. In both GPSR and LRS, it is easy to see that the packages from the chosen nodes (blue nodes in upper right corner of Figure 4a–f) will enter the hole region and consequently get stuck in the node of the concave region of the hole. At the time the GPSR switches to perimeter routing mode, packets arriving at the trapped node will be routed through the perimeter of the hole. As depicted in Figure 7b,e, this repetitive behavior can lead to an increase in the size of the hole region, as the edge nodes will receive larger amounts of packets during the forwarding process.

Face routing strategies tend to route data packets along the boundary of holes. Furthermore, approaches based on the one-way rule generate overhead for nodes located at one of the edges of the hole, since they cannot avoid forwarding data packets along the boundary of a hole. On the other hand, the LRS algorithm does not use the perimeter routing technique, but as we can see in Figure 7a,d, packets are also forwarded to the stuck node and subsequently routed around the hole toward the sink. Differently, from both LRS and GPSR, the PAtCH algorithm avoids the region of holes, creating alternative paths for flowing data to the sink, as shown in Figure 7c,f. This enhancement solves the problem of the energy depletion of the nodes at the edge of the hole and still optimizes the lifetime of the network.

## 5. Applicability of the Proposed Solution

The local minimum phenomenon is widely discussed in [10,11,51] and is characterized by the existence of an area between neighbors in which regular nodes cannot send out their data packets. Such a phenomenon is common for most of the geographic routing protocols and occurs in the presence of routing holes, which consist of a region where either the nodes are not available, or the available nodes cannot participate in the actual routing of the data due to various possible reasons. Routing holes can cause the interruption of the packet-forwarding process. Thus, hole detection and its bypass must be taken into account.

In this work, we consider that the sink node can communicate with the entire network because it is equipped with a long-range communication device. This sink can send a query packet to all regular nodes in a single hop. This is a relevant consideration in scenarios such as the ATTO project (Amazonian Tall Tower Observatory), in which we intend to use and improve this communication architecture. Reply packets from deployed nodes are routed to the sink using safe multi-hop paths computed by our novel location-free routing technique that uses the RSSI (Received Signal Strength Indicator) values to improve better routes and avoid hole regions during the routing process. Thus, our solution would be able to avoid holes and forward the information in multiple paths created by our mechanism.

Differently from GPSR, the PAtCH algorithm does not need to initiate a flood to create a planar graph. Excessive flooding is unsuitable for environments with poor communication quality. In all experimented scenarios, the PAtCH algorithm has the best performance evaluation. Despite being influenced by the RSSI inaccuracy, our novel geographic technique proved effective in avoiding routing holes without the need to perform additional calculations or extra loops. Our routing mechanism can be applied to mobile and ad hoc wireless networks, including IoT (Internet of Things) applications since there is no need for constant updating of node positioning information.

## 6. Conclusions and Future Work

In this paper, we proposed a novel location-free geographic routing algorithm called PAtCH that can avoid hole regions in Wireless Sensor Networks. Our approach considers that the sink node has a large communication capacity, being able to reach all network nodes in a long-range single hop. The RSSI values of the nodes are used to choose the neighbor geographically closest to the sink (final destination). Our PAtCH algorithm can forward packets around a hole region without entering such areas, thus contributing to mitigate the energy hole problem in WSNs.

A set of experiments was performed to evaluate our PAtCH algorithm. PAtCH outperforms GPSR (Greedy Perimeter Stateless Routing) by not using position information of the nodes and yet still being able to provide effective greedy paths toward the sink. We have shown that it is possible to avoid routing paths converging into hole regions. With this improvement, the nodes located in areas with holes will not be intensively used during message forwarding, optimizing the energy consumption in such areas. Our results show the benefits introduced by our location-free geographic routing approach to avoid hole regions in WSNs.

Our results are promising, but some limitations need to be overcome, such as the establishment of a limit for the size of the network and the implementation of an energy model compatible with the multi-purpose approach. As a future work, our objective is to evaluate our proposed approach in the context of multiple holes and adapt its capabilities to the Wi-Fi direct protocol [52], which has the ability to provide intercommunication between mobile devices without the requirement of a wireless access point, thereby offering data rates of up to 250 Mbps, which is a measure that is adequate for several mobile applications. We also plan to evaluate our PAtCH algorithm by implementing a single-hop feature of the sink node by using multi-hop communication in scenarios without a powerful communication device.

## Figures and Tables

**Table 1:** Classification of the discussed algorithms.

	Does Not Rely on Location Systems	Performs Multiple Forwarding Routes	Avoid Holes during Data Delivery
GPSR-Classic [10]		*√*	
RNGHAR [22]			*√*
HPS [29]			*√*
BVR-VCM [34]		*√*	
Approximate-Polygon [35]			*√*
HA-ECMAE/2H [26]			*√*
CCBGR [27]			*√*
GPSR-Improved [28]		*√*	
SGFTEM [32]			*√*
PPO-Based [33]			*√*
LRS [36]	*√*		
**PAtCH**	*√*	*√*	*√*

**Table 2:** Default scenario configuration.

Parameter	Value
Sensor Filed (grid size)	200 m × 200 m${}^{2}$
Number of Nodes	512 (disturbed grid)
Number of Sinks	1
Density	0.03 nodes/m${}^{2}$
Sink Location	Lower left corner
Default Communication Range	30 m
Short RSSI inaccuracy	10% of real distance
Long RSSI inaccuracy	1% of real distance

## Data Availability

The data are not publicly available due to company policy.

## Abbreviations

The following abbreviations are used in this manuscript:

ATTO	Amazonian Tall Tower Observatory
GPSR	Greedy Perimeter Stateless Routing
GPS	Global Positioning System
RSSI	Received Signal Strength Indication
PAtCH	Proactive Approach to Circumvent Holes in Wireless Sensor Networks
WSN	Wireless Sensor Networks
